# Physiological role of actin regulation in male fertility: Insight into actin capping proteins in spermatogenic cells

**DOI:** 10.1002/rmb2.12316

**Published:** 2020-01-22

**Authors:** Tetsuji Soda, Yasushi Miyagawa, Shinichiro Fukuhara, Hiromitsu Tanaka

**Affiliations:** ^1^ Department of Urology Osaka University Graduate School of Medicine Suita Japan; ^2^ Department of Urology Osaka Police Hospital Osaka Japan; ^3^ Department of Urology Sumitomo Hospital Osaka Japan; ^4^ Faculty of Pharmaceutical Sciences Nagasaki International University Sasebo Japan

**Keywords:** actin, actin cytoskeleton, CapZ actin capping protein, male infertility, spermatogenesis

## Abstract

**Background:**

During spermatogenesis, cytoskeletal elements are essential for spermatogenic cells to change morphologically and translocate in the seminiferous tubule. Actin filaments have been revealed to be concentrated in specific regions of spermatogenic cells and are regulated by a large number of actin‐binding proteins. Actin capping protein is one of the essential actin regulatory proteins, and a recent study showed that testis‐specific actin capping protein may affect male infertility.

**Methods:**

The roles of actin during spermatogenesis and testis‐specific actin capping protein were reviewed by referring to the previous literature.

**Main findings (Results):**

Actin filaments are involved in several crucial phases of spermatogenesis including acrosome biogenesis, flagellum formation, and nuclear processes such as the formation of synaptonemal complex. Besides, an implication for capacitation and acrosome reaction was also suggested. Testis‐specific actin capping proteins are suggested to be associated with the removal of excess cytoplasm in mice. By the use of high‐throughput sperm proteomics, lower protein expression of testis‐specific actin capping protein in infertile men was also reported.

**Conclusion:**

Actin is involved in the crucial phases of spermatogenesis, and the altered expression of testis‐specific actin capping proteins is suggested to be a cause of male infertility in humans.

## 1 INTRODUCTION

Spermatogenic cells change their shape dramatically during spermatogenesis,^1^ and a tremendous number of proteins and molecules are involved in each phase. Cytoskeletal elements are essential for morphological roles or the translocation of spermatogenic cells to move from the base of the seminiferous tubule toward the luminal edge during spermatogenesis.^2^, ^3^ The eukaryotic cytoskeleton is composed of microtubules, intermediate filaments and actin filaments (microfilaments), and each of these elements is fundamental to eukaryotic cell biology and integral to a diversity of cellular functions.^4^ Actin filaments, one of the fundamental components of the cytoskeleton, have been revealed to be concentrated in specific regions of both spermatogenic cells and Sertoli cells and to serve as a structural scaffold and track for motor proteins.^5^ In Sertoli cells, there are specialized actin‐containing structures involved in basal and apical ectoplasmic specialization (ES), which are composed of actin filament bundles sandwiched in between cisternae of endoplasmic reticulum and the opposing plasma membranes of the spermatid.^6^ Basal ES is known as a component of the blood‐testis barrier,^7^ and apical ES is a kind of component of cell‐cell anchoring junctions between Sertoli cells and spermatogenic cells.^8^, ^9^ However, actin was also reported to exist in organellae of spermatogenic cells such as the acroplaxome and the manchette.^10^, ^11^ The acroplaxome is an actin‐containing plate that connects the acrosome and the nuclear envelope of the spermatid.^12^ The manchette is a temporary structure located at the caudal part of the acrosome and disappears when the nucleus completes morphogenesis.^13^ The manchette consists of microtubules and actin filaments, and so‐called intramanchette transport (IMT) provides materials such as some structural and functional molecules for nuclear shaping and tail formation.^11^, ^14^ These prerequisite functions of actin filaments during spermatogenesis are sustained by a large number of actin‐binding proteins such as Eps8, Arp2/3, formin, and paladin.^15^, ^16^, ^17^, ^18^ Actin capping protein, which is one of the most important actin‐binding proteins, has recently been found to be related to male infertility in human.^19^ In this review, we focus on the role of actin filaments in spermatogenic cells and provide insight into testis‐specific actin capping protein along with some speculation on its role in spermatogenic cells.

## 2 STRUCTURES OF ACTIN FILAMENTS AND ITS ROLE IN SPERMATOGENIC CELLS

**Figure 1:**
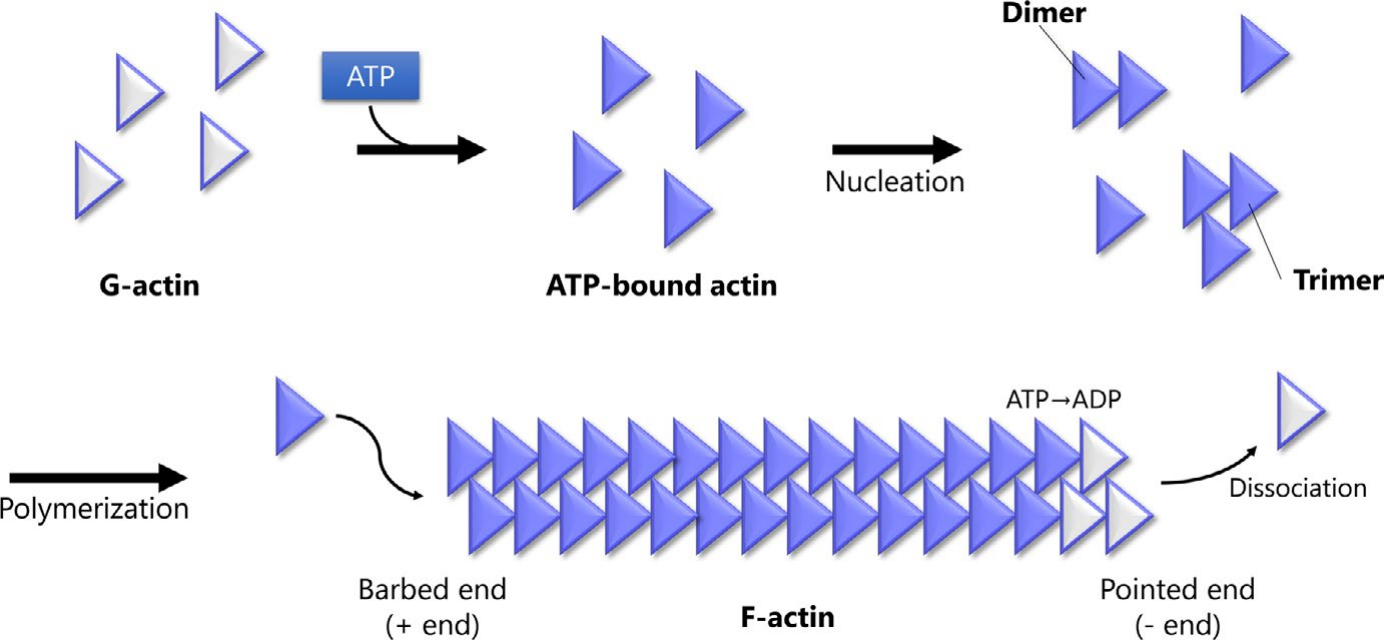
Dynamics of actin filament polymerization. In the formation of actin filaments, globular actin (G‐actin) binds to adenosine triphosphate (ATP), forms stable di‐ or trimers, and, finally, the filaments elongate by the addition of monomers. Actin filaments are polar because the subunits in the filament all point in the same direction. They have a fast‐growing barbed end (plus end) and a slow growing or dissociating pointed end (minus end). Spontaneous hydrolysis of ATP and the dissociation of phosphate destabilize the filament and induce the release of G‐actin. Among over 100 accessory proteins of actin, capping proteins bind to the barbed end of actin filaments and regulate the assembly and turnover of actin. ADP, adenosine diphosphate; ATP, adenosine triphosphate

The actin filament is highly conserved across a diverse set of eukaryotic species.^20^ Under physiological conditions, actin monomers, called globular actin, spontaneously polymerize into long stable filaments, filamentous actin (F‐actin), with a helical arrangement of subunits.^21^ In the formation of actin filaments, globular actin binds to ATP, forms stable di‐ or trimers, and, finally, the filaments elongate by the addition of monomers (Figure 1).^22^ Actin filaments are polar because the subunits in the filament all point in the same direction. They have a fast‐growing barbed end (known as the plus end) and a slow growing or dissociating pointed end (known as the minus end).^23^ Over 100 accessory proteins are used to maintain a pool of actin monomers, initiate polymerization, restrict the length of actin filaments, regulate the assembly and turnover of actin filaments, and crosslink filaments into networks or bundles.^22^, ^24^ Dynamic actin filament networks are required for numerous functions related to cell shape and movement, such as migration, contraction, adhesion, and protrusion.^25^

**Figure 2:**
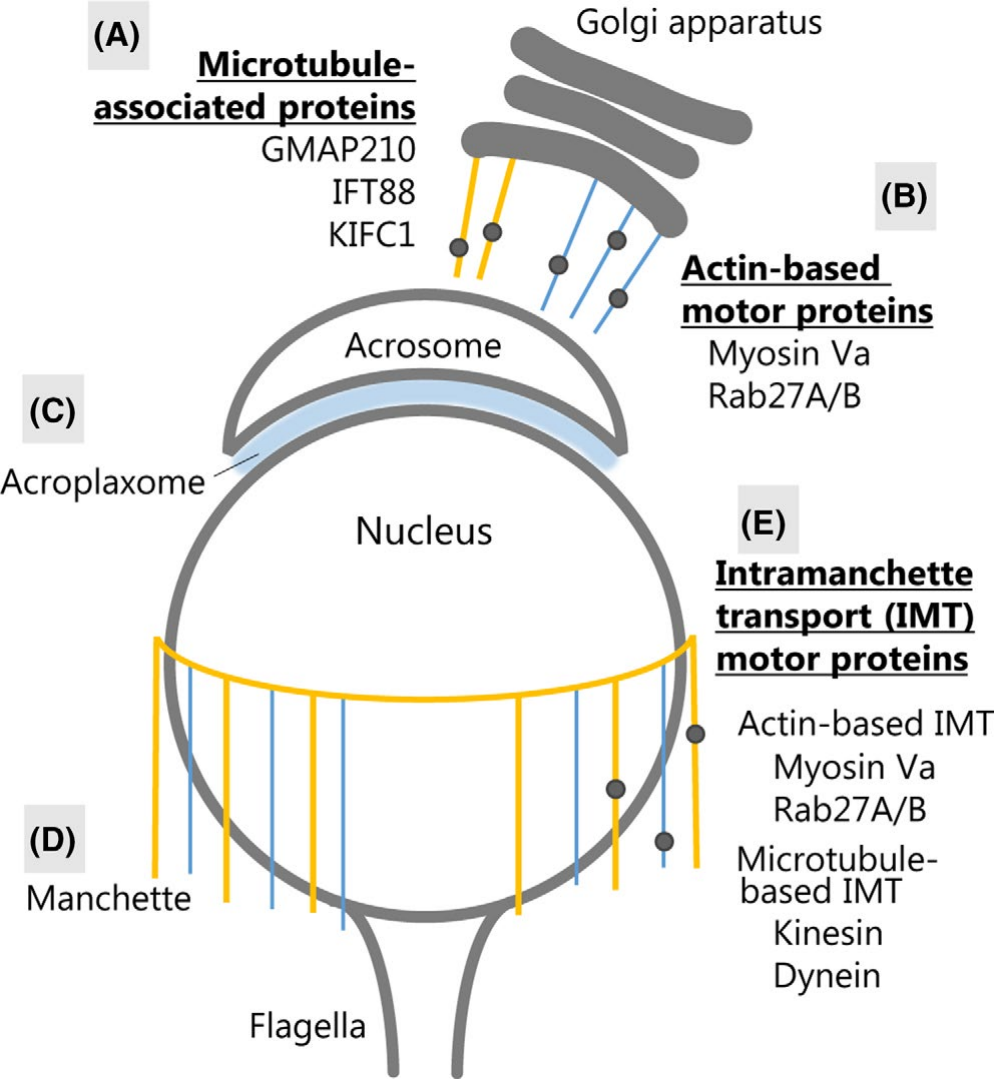
Diagrammatic representation of actin‐containing cytoskeletal structures including the acrosome biogenesis vesicle pathway and intramanchette vesicle pathway. The vesicles transported along either actin filaments or microtubules are symbolized by black points. Actin filaments and microtubules are shown by blue and yellow lines, respectively. (A) Proacrosomal vesicles derived from the Golgi apparatus are transported to the developing acrosome along actin filaments or microtubule tracks. Microtubule‐associated golgin GMAP210, IFT88, and KIFC participate in acrosome biogenesis. (B) Actin‐based motor proteins myosin‐Va and Rab27A/B complex transport the proacrosomal vesicles to the developing acrosome. (C) The acroplaxome is sandwiched between the acrosome and the nucleus and anchors the acrosome to the nucleus. (D, E) The manchette consists of the microtubule and F‐actin. It is hypothesized that proteins are transported to the base of flagella through the manchette by intramanchette transport (IMT) and to elongating flagella by intraflagellar transport (not depicted)

During spermatogenesis, spermatogenic cells undergo morphological changes that are classified into many phases, such as condensation of the sperm head, acrosome formation, elongation of the tail, and mitochondria translocation.^26^, ^27^ Acrosome biogenesis is one of the earliest events in spermiogenesis. Proacrosomal vesicles derived from the Golgi apparatus or from the endocytic pathway are transported to the developing acrosome^28^, ^29^, ^30^ along actin filaments^31^ and microtubule tracks.^32^ Actin‐based motor proteins myosin‐Va and Rab27A/B^33^, ^34^ and microtubule‐associated proteins such as GMAP210, IFT88,^35^ and KIFC1^32^ were suggested to participate in proacrosomal vesicle transport and biogenesis of the acrosome‐acroplaxome complex (Figure 2A,B).^35^ The acroplaxome is an F‐actin‐keratin 5‐containing cytoskeletal plate that anchors the acrosome to the spermatid nucleus (Figure 2C).^12^ Disruption of F‐actin by cytochalasin D results in nuclear‐acrosome detachment and disruption of the expanding edge of the acrosome.^5^

The manchette is formed after microtubules increase around the nucleus (Figure 2D).^36^ Its existence is transient as it is formed in early spermatids and completely dissolves by the time mature sperm are formed.^37^ The manchette is hypothesized to be involved in nuclear shaping and its improper positioning to be a cause of abnormal formation of the nucleus.^38^ The placement of the manchette along the nucleus is suggestive of a role for this structure in the redistribution of cytoplasmic contents necessary for their removal prior to spermiation.^39^ Both IMT and intraflagellar transport (IFT) are similar types of molecular transport and are suggested to involve molecular motors mobilizing a multicomplex protein raft to which cargo proteins or vesicles are linked (Figure 2E).^40^, ^41^ During IFT, precursors for the assembly of the axoneme of a flagellum or a cilium are transported to the assembling tip of the axoneme by kinesin‐II. In contrast, both microtubules and actin filaments of the manchette support IMT. Along with the microtubule‐based motors kinesin and dynein that are resident on the manchette, the actin‐based molecular motor myosin‐Va is also found in the acroplaxome and in the manchette of developing spermatids.^42^, ^43^

In the flagella, actin filament is observed in the midpiece around the mitochondrial sheath in a double‐helix structure.^44^ The actin cytoskeleton is speculated to be involved in the migration of mitochondria to the midpiece during spermiogenesis and in providing a scaffold that confines mitochondria in this cellular compartment. Besides, actin filament is also distributed throughout the principal piece, forming short bundles. Spectrin, which is a widespread structural actin‐associated protein^45^ and acts as a molecular spring and dramatically alters the elasticity of actin,^46^ is localized in both pieces and is suggested to provide the flagella with required elasticity during sperm hyperactivation.^44^

Other than in its morphological role in spermatogenic cells, actin polymerization also correlates with sperm capacitation in different mammalian species.^47^, ^48^ It has also been proposed that F‐actin remodeling occurs during the acrosome reaction.^49^, ^50^ In the presence of cytochalasin D, an inhibitor of actin polymerization, there was a marked decrease in the fertilizing capacity of boar spermatozoa.^47^ Cytochalasin D or anti‐actin monoclonal antibody inhibited the zona pellucida‐induced acrosome reaction in human sperm.^51^, ^52^ Actin dynamics are thus suggested to play a role in sperm function. Furthermore, actin may be involved in many nuclear processes such as the formation of the synaptonemal complex (SC), which is a protein structure formed between homologous chromosomes and functions to zipper the two homologs. During the prophase of meiosis, homologous chromosome pairing and recombination are facilitated by SC.^39^ Actin dynamics may rely on the formation of SC, as the depletion of ALKBH4, which is a modulator of specific actin‐myosin dynamics, leads to the insufficient establishment of SC.^53^ Studies of long‐range interphase chromosome movements in mammalian somatic cells show dependency on nuclear actin and myosin.^54^ There may be a network of both nuclear and cytoplasmic actin interactions in nuclear processes.^55^, ^56^

## 3 ACTIN CAPPING PROTEIN

The dynamics of the actin filament system in non‐muscle cells are regulated by actin‐binding proteins that can be divided into distinct groups.^57^, ^58^, ^59^ Capping proteins bind to one of the ends of actin filaments and influence subunit reactions there, and they were divided into three families: (a) gelsolin and villin, (b) fragmin/severin, and (c) a group termed simply “capping protein”.^58^, ^60^ The gelsolin and villin family have been found in vertebrates. The proteins in this family are monomers of 90‐95 kDa that also require calcium and are sometimes isolated as a 1:1 complex with actin.^61^ The fragmin/severin and capping protein families may be universal in their distribution as they have been isolated from protozoa and vertebrates. The proteins in the fragmin/severin family consist of polypeptides of about 45 kDa, which are often isolated as a 1:1 complex with actin. They require calcium to cap, nucleate, and sever.^62^ Capping protein, referred to hereafter as CP, caps the barbed ends of actin filaments with high affinity,^63^ thereby preventing the addition or loss of actin subunits.^64^ CP is an α/β heterodimer with an α subunit of 32‐36 kDa and a β subunit of 28‐32 kDa.^60^ Individual subunits are unstable, but the heterodimer is very stable.^65^ The α and β subunits require each other for actin‐binding activity in vitro and stability in vivo.^66^, ^67^ The mechanism of CP binding to the barbed end of the actin filament was previously reviewed.^68^, ^69^ The complex behaves as a single protein in terms of its physical properties.^70^

The CP molecule has the shape of a mushroom.^71^ Although there is no similarity in the sequence of each of its subunits, the two subunits have very similar secondary and tertiary structures.^71^ No other protein structures in the Protein Data Bank resemble the CP structure.^72^ Phylogenetically, when comparing the individual subunits in different organisms, sequence similarity of CP is much higher than other actin‐binding proteins. BLAST searches readily reveal apparent homologs of both subunits in vertebrates, invertebrates, plants, fungi, insects, and protozoa.^73^ The sequences of the β subunits appear to be more strongly conserved than those of the α subunits.^65^ Organisms other than vertebrates have single genes encoding each of the CP subunits. In contrast, vertebrates have two somatically expressed isoforms of each subunit, termed α1/α2 and β1/β2, and one additional set of male germ cell‐specific isoforms, α3 and β3.^74^, ^75^, ^76^, ^77^ The isoforms of the α subunits are encoded by different genes, whereas those of the β subunits are produced from a single gene by alternative splicing.^74^ The sequences of both the α1 and α2 and β1 and β2 isoforms are conserved across vertebrates, suggesting that they have distinct functions in vertebrates. Little evidence exists regarding specific functions of the α isoforms, but they are expressed at varying ratios in different cells and tissues.^75^ The β1 and β2 isoforms could not be substituted for each other in muscle cells, thus supporting the hypothesis that they have distinct functions.^78^

In muscle cells, CP is an essential component of the Z‐disk, where it caps the barbed ends of actin‐based thin filaments.^79^, ^80^ In non‐muscle cells, CP is important for the assembly of cortical actin and for cases of actin‐based motility, such as the formation of membrane protrusions at the leading edge of migrating cells.^72^ CP regulates the actin‐related protein 2/3 (ARP2/3) complex‐dependent actin assembly at various cellular membranes,^81^, ^82^ including lamellipodial protrusions, adherens junctions, and at sites of podosomes and invadopodium formation and phagocytosis and micropinocytosis. CP is also associated with endosomal compartments that undergo fission and fusion.^79^ Recent evidence has suggested that CP also regulates the assembly of actin filaments in filopodia,^83^ which can arise from dendritic actin networks.

## 4 TESTIS‐SPECIFIC ACTIN CP

Germ cell‐specific CPs named CPα3 and CPβ3 are expressed in mammalian testis. It was first revealed that a new CP α subunit gene, other than the somatic CP α1 or α2 gene, was cloned from a complementary DNA (cDNA) library generated by subtracting messenger RNA derived from mutant (W/W^v^) testis from wild‐type testis cDNA. The new α subunit gene was named mouse germ cell‐specific gene 3 (*gsg3*)^84^ and was later referred to as CPα3 (*cpα3*).^85^ Genomic analysis has revealed that mouse *cpα3* is an intronless gene on chromosome 6.^86^, ^87^ The expression of *cpα3* is haploid germ cell‐specific, and CPα3 protein expression coincides with the position of the developing acrosome in the rat testis.^77^ The subcellular localization of CPα3 in mouse sperm changes dynamically from the flagellum to the postacrosomal region of the head during epididymal maturation.^88^ Besides, CPα3 shows dynamic changes during the acrosome reaction in bovine sperm.^89^ *cpα3* cDNA was identified in human as an orthologue of the mouse *cpα3*. The messenger RNA of the human *cpα3* gene was expressed exclusively in testis as was mouse *cpα3.* 90 Therefore, it has been suggested that CPα3 is one of the actin regulators that may play a critical role in spermatogenesis and sperm function.

**Figure 3:**
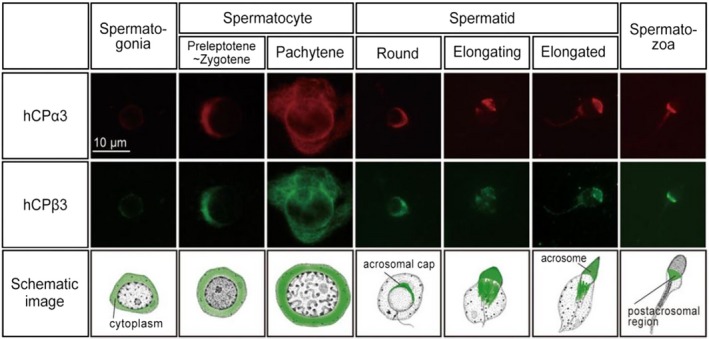
Protein expression profiling of human CPα3 and CPβ3 during human spermatogenesis. Cell diagrams indicating the steps of spermatogenesis are modified from Walker, 2010. The localization of human CPα3 and CPβ3 was almost identical and dynamically changed from cytoplasm to the acrosomal cap, acrosome, and postacrosomal region of the mature sperm head

In contrast, CPβ3, which is considered to be a heterodimeric counterpart of CPα3, was first reported in bovine and later in mouse.^76^, ^91^ Recently, human CPβ3 was reported to be expressed in human testis.^19^ The localization of human CPβ3 was completely identical to that of human CPα3 and changed dynamically during spermatogenesis (Figure 3).^19^ Especially, the cellular localization migrated from cytoplasm to the acrosomal cap and acrosome during spermatid maturation, which is called spermiogenesis. Subsequently, human CPβ3 accumulated in the postacrosomal region of the head in mature spermatozoa. Although the physiological role of testis‐specific CP during spermatogenesis is not clarified yet, the dynamic change of CPα3 and CPβ3 localization may be associated with the biogenesis of the acrosome and manchette of the head as those organelles contain actin filaments.^11^, ^12^

Besides its role in spermiogenesis, CPα3 shows a dynamic pattern of localization during capacitation and the acrosome reaction in mature mouse sperm.^92^ CPα3 localizes to the anterior acrosome before capacitation and presents more diffuse patterns after capacitation. Shortly after the induction of the acrosome reaction, CPα3 redistributes to the postacrosomal compartment and finally diminishes when the acrosome reaction is completed. The fact that actin polymerization occurs during capacitation and is subsequently reduced or lost from the acrosomal region after the acrosomal reaction in mouse spermatozoa^49^, ^50^, ^51^, ^93^ may be associated with the dynamic pattern of CPα3.^92^

## 5 ASSOCIATION OF TESTIS‐SPECIFIC ACTIN CP AND MALE INFERTILITY

**Figure 4:**
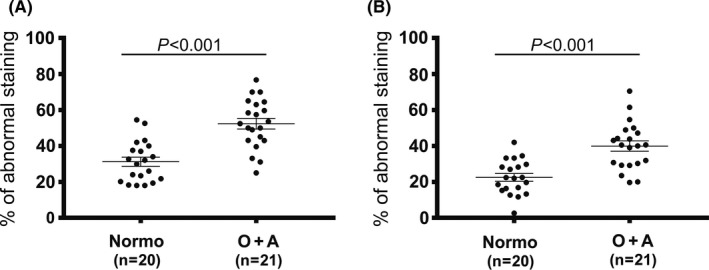
Comparison of sperm abnormally stained by anti‐CPα3 or anti‐CPβ3 antibodies between male volunteers with normozoospermia (Normo group, n = 20) and infertile men with oligozoospermia and/or asthenozoospermia (O + A group, n = 21). The horizontal bars represent the mean ± SEM. (A) The percentage of abnormally stained sperm was significantly higher in the O + A group (52.4 ± 3.0%) than in the Normo group (31.2 ± 2.5%) (*P* < .001). (B) The percentage of abnormally stained morphologically normal spermatozoa selected by David's criteria was still higher in the O + A group (39.9 ± 2.9%) than in the Normo group (22.5 ± 2.1%) (*P* < .001)

Considering the testis‐specific expression of CPα3 and β3 during spermatogenesis and capacitation or the acrosome reaction, it is not surprising that the lack of function of these proteins is associated with male infertility to some extent. The N‐ethyl‐N‐nitrosourea‐induced mutant mice with the *cpα3* gene failed to remove excess cytoplasm during spermiation.^91^ Mutation in the *cpα3* gene is suggested to lead to the disruption of F‐actin in condensing spermatids and may result in defective function of the tubulobulbar complex through which excess cytoplasm is taken up by Sertoli cells.^94^, ^95^ In human, alteration of immunostaining of CPα3 and β3 in a male infertile population possibly because of protein modification or degradation was shown by the comparison of sperm between men with normal semen analysis and infertile men with oligozoospermia and/or asthenozoospermia (Figure 4A).^19^ Furthermore, even in the comparison of morphologically normal spermatozoa, abnormal immunostaining was still higher in the infertile men (Figure 4B). These results may imply that human testis‐specific CPs are important not only for normal spermatogenesis but also for some unknown sperm function. In high‐throughput sperm proteomics using normozoospermic samples with different in vitro fertilization outcomes (pregnancy vs no pregnancy), human CPα3 was identified as one of the less abundant proteins in sperm.^96^ However, evidence from single nucleotide morphism analysis of the *cpα3* gene between fertile and infertile men indicates that the *cpα3* gene may not be a genetic factor for male infertility.^97^ In mammals, the *cpα3* gene is located back‐to‐back with the phospholipase C isoform ζ (PLCζ) gene.^98^ PLCζ is considered as a nominee for sperm‐associated oocytes activating factors and to induce triggering of Ca2 + oscillations.^99^, ^100^ These two genes share a common bidirectional promoter with a putative cAMP‐responsive element modulator of protein recognition sites,^77^, ^90^ and individuals with low or failed fertilization showed significantly lower expression of these two genes.^98^ Human CPα3 was suggested to be indirectly associated with oocyte activation. Further investigation is needed to specify the reasons for the low expression of human testis‐specific CPs in infertile men.

## 6 CONCLUSION

Actin fibers are involved in several crucial phases of spermatogenesis, such as acrosome biogenesis, flagellum formation, and nuclear processes. Furthermore, research has suggested an implication for capacitation and acrosome reaction. Such actin dynamics are regulated by actin‐binding proteins, and testis‐specific CP is one of the important actin‐binding proteins in spermatogenic cells. A lack of function of testis‐specific CPs is associated with male infertility in mouse and human. Testis‐specific CPs have been shown to be associated with the removal of excess cytoplasm during spermatogenesis or oocyte activation after fertilization. Altered protein expression of testis‐specific CPs was suggested to be a cause of male infertility in human. Further examination is still needed to fully elucidate the function of testis‐specific actin CP.

## DISCLOSURES

*Conflict of interest:* The authors declare no conflict of interest. *Human and animal rights:* This article does not contain any study with human and animal participants performed by any of the authors.
